# Novel Pathological Mechanisms Revealed by Spatial Transcriptomic Analysis of Hippocampus in Aged Control, Primary Age-Related Tauopathy, and Alzheimer’s Disease

**DOI:** 10.21203/rs.3.rs-7303622/v1

**Published:** 2025-08-22

**Authors:** Hong-Wen Deng, Yun Gong, Qi-Lei Zhang, Di Wu, Anqi Liu, Tianying Li, Zhengwu Xiao, Yisu Li, Mohammad Haeri, Russell Swerdlow, Yiping Chen, Xiaoxin Yan, Hui Shen, Hong-Mei Xiao

**Affiliations:** Tulane University; Tulane University; Department of Anatomy and Neurobiology, Xiangya School of Medicine, Central South University; Tulane University; Tulane University; Central South University; Central South University; Tulane University; University of Kansas Medical Center; University of Kansas; Tulane University; Department of Anatomy and Neurobiology, Central South University Xiangya Medical School; Tulane University; Central South University

## Abstract

While both Primary Age-Related Tauopathy (PART) and Alzheimer’s Disease (AD) involve the accumulation of hyperphosphorylated tau (pTau)-positive neurofibrillary tangles (NFTs) in the hippocampus, PART is distinguished by the absence of β-amyloid (Aβ) deposition and is generally associated with milder cognitive impairment than AD. To delineate cellular and molecular mechanisms that are common or uniquely linked to disease progression in PART and AD, we constructed a transcriptome-wide, high-resolution atlas of the human hippocampus using samples from six individuals spanning the aged control (AC), PART, and AD groups. Our results supported that PART represent a precursor stage of AD, as evidenced by the altered transcriptional profiles of excitatory neurons (Exc) in the PART group, which exhibited a markedly increased capacity to promote Aβ production compared to both AC and AD groups. While the microglia (Mic) were reactivated in the PART group, this response was reduced in AD samples despite the presence of Aβ deposition, and appeared to further induce NFTs formation as a loop consequently driving the progression from PART to AD. Furthermore, subregion interactions in the signalling pathways related to neuronal survival and the maintenance of blood-brain-barrier (BBB) integrity were decreasing in the PART and disrupted in the AD groups, compared to the AC group. Additionally, we found a P53 signalling-related gene, *TP53INP2*, was uniquely upregulated in astrocytes near large vessels in AD. This suggests a potential mechanism of vessel-induced neuronal apoptosis in AD, a feature absent in AC and PART. In summary, our study offers new insights into the relationship between PART and AD, along with the molecular mechanisms driving the transition from PART to AD. Furthermore, we identified key molecular pathways associated with BBB disruption and vascular-associated neuronal degradation in AD which were absent in PART. These findings deepen our understanding of AD pathogenesis and may inform the development of targeted therapeutic strategies.

## INTRODUCTION

Alzheimer’s disease (AD) is a neurodegenerative disorder primarily defined by its onset with memory deficits and cognitive difficulties, progressively extending to affect behavior, language, spatial perception, and motor functions (1–3). As of 2024, around 6.9 million Americans aged 65 and older are affected by AD, and this number is projected to nearly double to 13.8 million by 2060 (4). Given the devastating impact of this disease, numerous studies have investigated its underlying mechanisms. These studies have identified various risk factors, including aging (5), cardiovascular health (6), education (7), diet (8), social interactions (9), brain injuries (10), and more than 70 genetic markers (11, 12). At the pathology level, AD brain is marked by the accumulation of extracellular amyloid-β (Aβ) plaques (13) and intracellular hyperphosphorylated tau aggregates as neurofibrillary tangles (NFTs) (14) in the gray matter. These pathological features can induce cytotoxicity, drive neuroinflammation, and impair mitochondrial function, which collectively contribute to neuronal stress, degeneration, and eventual brain atrophy (13, 15). Despite these insights, the pathological mechanisms underlying AD remain unclear, limiting the development of effective medical interventions. Although several drugs targeting the management of this disease, particularly those aimed at clearing Aβ plaques, have been approved by the FDA, these therapeutic approaches have largely failed in clinical trials due to adverse side effects or insufficient efficacy (16). Thus, more comprehensive studies utilizing cutting-edge technologies are essential to deepen our understanding of this devastating disease and to develop more effective therapeutic strategies.

Employing the cutting-edge technology of spatial transcriptome (ST) (17), several studies investigated different brain regions, including prefrontal cortex (PFC) and middle temporal gyrus (MTG), uncovering novel insights into the pathological mechanisms underlying AD (18–20). In addition to these regions, the hippocampus is also an important brain area of progressive pathology in AD and merits detailed spatial investigation. As a critical structure in the medial temporal lobe responsible for memory and cognition, the hippocampus is among the earliest brain regions affected by AD–related neurodegeneration (21), offering an important opportunity to investigate early molecular changes associated with this disease. Additionally, the hippocampus serves as a valuable model for studying Primary Age-Related Tauopathy (PART), a neurodegenerative condition characterized by tau protein accumulation in the medial temporal lobe in the absence of significant amyloid-beta (Aβ) deposition, and commonly observed in aging individuals (22). Compared to AD, PART is associated with less neuronal loss and typically results in milder cognitive impairment. Although the filament structure of the NFTs in PART and AD are similar (23), it remains unclear whether PART represents an early histopathological stage of AD or simply the products of normal brain aging (24). Thus, understanding the pathological divergence among aged control (AC), PART, and AD could yield critical insights into the shared and unique molecular mechanisms underlying PART and AD, as well as the molecular events that drive Aβ accumulation and neuronal degeneration in AD. These insights may provide a strong foundation for developing future therapeutic strategies against this devastating disease. While two studies (25, 26) have investigated ST in the human hippocampus for AD, one did not include individuals with PART (26), and the other used the image-based GeoMx platform (25), which may not fully capture pathological molecular alterations between PART and AD due to its relatively low sensitivity compared to sequencing-based platforms (27). Therefore, studies using high-sensitive, sequencing-based ST platforms on hippocampal tissue from individuals with AC, PART, and AD are needed to comprehensively delineate the molecular differences among these conditions.

In this study, we employed the 10x Genomics Visium ST platform to construct an unbiased transcriptional atlas of the human hippocampus across AC, PART, and AD groups. Our goal was to uncover pathological molecular alterations among these groups, identify potential links between PART and AD, and explore the mechanisms underlying neuronal degeneration in AD. This high-resolution approach revealed transcriptomic signatures indicating that PART may represent a transitional stage from AC toward AD. In PART, upregulated transcripts in Exc appeared to promote Aβ production. Mic reactivation was already enhanced in PART to clear excessive Aβ. However, this microglial response was diminished in AD, potentially contributing to Aβ accumulation, NFT formation, and the progression from PART to AD. Moreover, inter-subregion support for neuronal survival and blood-brain barrier (BBB) integrity was reduced in PART and nearly absent in AD. Furthermore, the BBB disruption in AD was associated with activation of apoptotic pathways in Ast located near large blood vessels, suggesting a critical mechanism worsening neuronal degeneration.

## RESULTS

**Figure 1:**
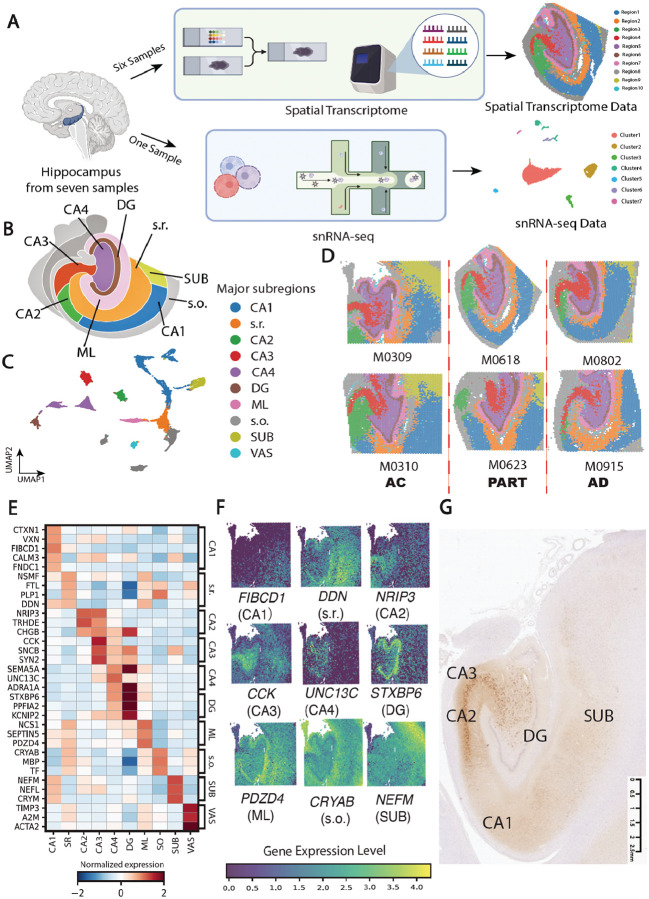
Spatially resolved transcriptomic profiles of the multiple subregions in human hippocampus (A) The analysis pipeline of the study. (B) Anatomical structure of the human hippocampus (C) The UMAP visualization of six subregion clusters across six samples. (D) The 10 hippocampal subregions identified across six samples in the AC, PART, and AD groups. (E) Heatmap of the marker genes in each subregion. The x-axis represents the subregions. Colors represent the normalized expression level of the gene in each subregion. (F) Scatter plots illustrating the expression levels of the subregion-specific gene markers across each hippocampal subregion. (G) The validation of FIBCD1 on human hippocampus using IHC.

Human Formalin-Fixed Paraffin-Embedded (FFPE) hippocampal tissue samples were collected from six individuals for ST analysis using the 10x Visium platform (Fig. 1A; **Fig. S1A-F**; Methods). The cohort included two AC (Braak stages I and II, Thal phase 0; two males, aged 88 and 79), two individuals with PART (Braak stage III, Thal phase 0; one male and one female, aged 87 and 81), and two AD patients (Braak stages VI and IV, Thal phase 3; one female and one male, aged 92 and 82; **Table S1**). Following the 10X Visium profiling, a total of 26,038 spots were captured across all six samples, with each spot detecting an average of 4,096 genes and 4,273 molecular counts. Previous studies on the human brain (28–30) have shown that Exc exhibited more prominent features compared to inhibitory neurons (Inh) and glial cells due to their larger size and essential roles in synaptic transmission and signaling pathways (31). To further validate the quality of our dataset, we aligned the ST data with the eosin-stained image from the same slide. The results demonstrated that spots with a high number of detected genes corresponded to regions enriched in Exc, such as the DG region (32, 33), supporting the high quality and reliability of our dataset for our comprehensive downstream data analysis (**Fig. S1A1-F1; Fig. S1A2-F2**).

### ST revealed unique gene expression patterns in distinct regions of human hippocampus

Conventionally, the human hippocampus is primarily composed of several soma-rich layer (SL) subregions, including stratum pyramidale (s.p.) of subiculum (Sub) and CA1–CA4, as well as the granule cell layer of dentate gyrus (DG). These areas are predominantly composed of Exc and play essential roles in information output, as well as in the integration and processing of neural signals (34). Surrounding the SL are distinct fiber layers (FL), which are organized above and below the cell body layers. These include the stratum oriens (s.o.), stratum radiatum (s.r.), and molecular layer (ML), which are primarily composed of dendrites, axons, and synaptic terminals. These fiber-rich regions are critical for synaptic input and output, serving as hubs for information integration and transmission (35). Furthermore, large vascular (VAS) structures are present across all subregions of the human hippocampus, supporting cellular oxidative metabolism and energy supply in these areas (36). To accurately delineate these spatial domains in our human hippocampal ST data, we applied a data-driven, unsupervised clustering algorithm, PRECAST (37), to group spatial spots into distinct domains based on their transcriptional profiles and spatial coordinates on the 10x Visium slides (Methods). We evaluated a range of spatial domain resolutions (k) and selected k = 15 based on the Bayesian Information Criterion (BIC) (**Fig. S2A**). Unsupervised clustering at this resolution grouped the spatial spots into 15 distinct clusters (**Fig. S2B**). To annotate these clusters, we mapped them onto the eosin-stained image and assigned anatomical labels based on their spatial positions (**Fig. S1A1-F1**). After grouping the clusters located within the same anatomical region, all clusters were annotated as 10 subregions, including SUB, CA1 to CA4, s.r., s.o., DG, ML, and VAS (Fig. 1B and 1C). Compared to previous ST studies using 10X Visium platform on human hippocampus (38, 39), which were unable to clearly distinguish certain regions, particularly CA2, CA3, and CA4, our data demonstrated a clear delineation of these regions. This may be due to their use of the 10x Visium platform on frozen sections of the human hippocampus, which are more prone to RNA degradation and increased background noise, including expression from non–protein-coding genes (40). These factors may hinder accurate distinction between hippocampal subregions. This result underscored the significant advantages of using the 10x Visium platform on FFPE-preserved human hippocampal tissue to construct a transcriptome atlas, enabling comprehensive and unbiased profiling of protein-coding genes.

We performed differential gene expression (DGE) analysis by comparing each subregion against all others, aiming to identify distinct transcriptional signatures for each hippocampal subregion (Fig. 1D; **Table S2**; Methods). Briefly, subregions enriched with Ex, such as the SUB, CA1 to CA4, and DG, showed high expression of genes associated with Exc functions. These include genes involved in neuronal calcium signaling (e.g., *CALM3, CCK, SYN2, CALB1*) and neuron synapse functions (e.g., *STXBP6, PPFIA2, SNCB*) (Fig. 1E). In contrast, subregions with a higher proportion of glial cells, including the s.r., s.o., and ML, displayed enrichment of genes related to myelin formation and maintenance (e.g., *PLP1, MBP*). Additionally, in the ML region, we observed upregulation of genes associated with neuronal dendrite function (e.g., *NCS1, SEPTIN5*) (41, 42), compared to other subregions. This is consistent with that the ML region is composed of neuronal dendrites originating from the DG (43). Furthermore, we performed Gene Ontology (GO) analysis on transcriptional markers to elucidate the distinct biological functions associated with each hippocampal subregion. GO terms associated with neuronal and synaptic functions, including “Chemical Synaptic Transmission”, “Neuron Development”, and “Axon Development”, were enriched in the SUB, CA1, CA2, CA3, and CA4 regions, which are predominantly composed of Exc. In contrast, GO terms related to myelin formation and cellular migration (e.g., “Myelination and Regulation of Cell Migration”) were highly enriched in SR and s.o., regions primarily consisting of glial cells, including Ast, Mic, and oligodendrocytes (Oli) (**Table S3**). The subregion-specific transcriptional profiles in our data are consistent with previous ST data from the human hippocampus (26), further supporting the accuracy of our clustering and annotation.

The intricate histological architecture of the hippocampus makes manual region annotation especially challenging, particularly for subfields such as CA1, CA2, CA3, and CA4, which lack clearly defined histological boundaries (44). This difficulty complicates the precise characterization of the unique biological functions of neurons and glial cells in these regions and hinders the study of their subregion-specific pathological changes during the early stages of AD. Therefore, identifying reliable biomarkers for each region is crucial for distinguishing these areas and capturing region-specific molecular dynamics of brain cells at the onset of AD. To address this, we identified subregion-specific marker genes based on the highest fold-change compared to their expression in other regions. These included *FIBCD1* for CA1, *DDN* for SR, *NRIP3* for CA2, *CCK* for CA3, *UNC13C* for CA4, *STXBP6* for DG, *PDZD4* for ML, *CRYAB* for SO, and *NEFM* for SUB (Fig. 1F). These hippocampal subregion-specific markers were validated through immunohistochemistry (IHC; **Fig. S2C-D; Fig. S3A-C**). Given the limited availability of markers to delineate the boundary between CA1 and CA2, and the absence of previous reports on *NRIP3* expression in the human hippocampus, we examined the distribution of the NRIP3 positive cells. We observed that NRIP3 was highly expressed in the CA2 subregion but shows low expression in CA1, forming a clear boundary between the two (Fig. 1G). This distinct expression pattern suggests that NRIP3 may serve as a reliable marker for distinguishing CA2 from CA1. Furthermore, although certain region-specific markers showed significant differential expression between the AC, PART and AD groups within their respective regions (**Fig. S4A**), these markers were still enriched in their corresponding subregions when DEG analyses were performed separately within the AC, PART, and AD groups (**Fig. S4B**). This suggested that these markers are robust and can serve as reliable references for regional identification across AC, PART, and AD conditions.

### Deconvolution analysis to enhance the resolution from spot level to single-cell resolution

Given that current 10X Visium platform cannot provide transcriptional data at the single-cell (sc) resolution, we applied the deconvolution methods to identify cell compositions and infer cell type-specific gene expression patterns within each 10X Visium spatial spot. We employed BayesPrism (45), a Bayesian algorithm that simultaneously infers cell-type proportions and their unique transcriptional profiles within each ST spot, without requiring snRNA-seq reference data from adjacent tissue. The snRNA-seq data from human hippocampal samples of AD cases and controls, published by Mathys *et al*. (46), was considered as the reference. Although the reference data from Mathys *et al*. were generated from Caucasian individuals and our ST data were derived from Asian donors, previous studies have reported that the core structural and functional features of the hippocampus are conserved between these populations (47, 48). Furthermore, we performed snRNA-seq on a hippocampal sample from an age-matched Chinese individual with AD (Fig. 1A; **Table S1**; Methods) and when merged with the data by Mathys *et al*., the transcriptomic profiles from the Chinese individual aligned well with the major clusters observed in the Caucasian dataset (**Fig. S5A**), suggesting minimal batch or population-related effects in hippocampal transcriptional profiles.

**Figure 2:**
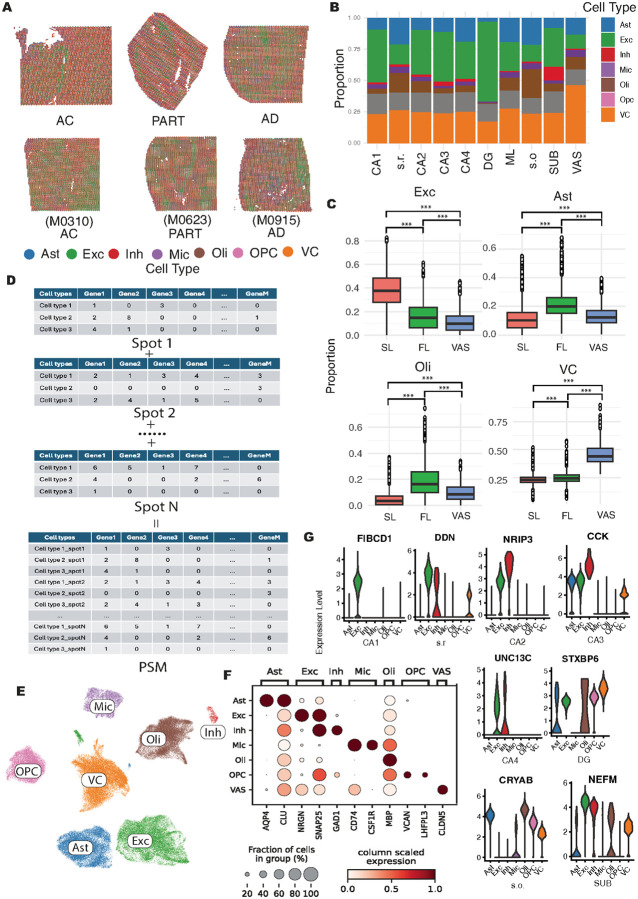
Spatial transcriptome deconvolution and cell type-specific transcriptional inference within each spatial spot (A) Spatial distribution of cell type composition in the hippocampus across AC, PART, and AD samples. Each spot represents a pie chart illustrating the proportional composition of cell types at that spatial location. (B) Bar plot of the cellular composition of major cell types across hippocampal subregions. Each bar represents the relative abundance of cell types within a given subregion. (C) The comparison of relative proportions of Exc, Ast, Oli, and VC in SL, FL, and VAS. The box plot represents the proportion of the specific cell type. N.S. indicates non-significant (adjusted P-value > 0.05) and *** represents adjusted P-value < 0.001. (D-F) The spearman correlation of the estimated cell type proportions from BayesPrism (x-axis) with those from CARD (D), SpaCet (E), and PANDA (F; y-axis) for Ast, Exc, Inh, Mic, Oli, OPC, and VC. Spearman correlation coefficients (ρ) and p-values are shown in each panel. (G) Mean Spearman correlation coefficients of cell type–specific transcriptional profiles from four permutation tests for BayesPrism and PANDA, respectively. The y-axis indicates the mean correlation coefficients. (H) Steps for pusedo-sc matrix construction. (I) UMAP visualization of the Ast, Exc, Inh, Mic, Oli, OPC, and VC at psedu-sc resolution (J) Expression levels of cell type–specific markers for Ast, Exc, Inh, Mic, Oli, OPC, and VC at pseudo–single-cell resolution. Dot size represents the proportion of cells expressing each marker, while color intensity indicates the expression level of the corresponding marker.

Through the deconvolution analysis, we assessed the proportion of each cell type within individual spots across distinct hippocampal regions (Fig. 2A–B). Exc were predominantly enriched in SL, including the SUB, CA1, CA2, CA3, CA4, and DG subregions (Fig. 2C; **Fig. S5B**). In contrast, Ast and Oli were largely localized to FL, such as the s.o., s.r., and ML. Notably, VC exhibited significantly higher proportions in the VAS region compared to both SL and FL These findings were consistent with previous studies (26, 39). Given that BayesPrism was originally developed to infer cellular compositions from bulk RNA-seq data rather than ST, we evaluated its performance on ST data by comparing its inferred cell compositions to those generated by deconvolution methods specifically designed for ST, including CARD (49), SpaCet (50), and PANDA (51). Across all cell types, BayesPrism results showed significant positive correlations with those from other methods (**Fig. S5C-E**), suggesting that BayesPrism, despite not incorporating spatial information, performs comparably to spatially aware deconvolution methods, supporting its reliability in this study. After estimating the cellular composition of each spatial spot, we inferred the corresponding cell type-specific transcriptional profiles (Methods). To evaluate the robustness of BayesPrism, we performed a permutation test by randomly splitting the snRNA-seq data into two groups, each containing half of the cells from every cell type. Each group was independently used as a reference in BayesPrism to infer cell type-specific gene expression profiles for each spot, and the inferred results were compared across runs to assess consistency. For comparison, we applied the same strategy using PANDA to benchmark BayesPrism’s performance. Since the mean correlation of gene expression levels across four permutation tests was higher using BayesPrism than PANDA (**Fig. S5F**), this result suggested that BayesPrism offers greater robustness in inferring cell type–specific gene expression in each spatial spot. Therefore, BayesPrism is currently the most suitable method for our study.

To enhance the ST data into sc resolution, we have constructed the spatial pseudo-sc matrix (PSM) data based on the inferred cell-type specific gene expression patterns within each spot (Fig. 2D). After removing the cells with fewer than 300 detected genes and genes that are expressed in fewer than three cells, we have captured 121,087 cells with median genes detected 621, which is similar to current cutting-edge ST platforms with sn resolution (26, 52–54). Following cell type clustering and visualization, the pseudo-cells were annotated as distinct major brain cell types (Ast, Exc, Inh, Mic, Oli, OPC, VC) (Fig. 2E). Marker genes for each pseudo-cell cluster were compared and found to be consistent with those reported in previous snRNA-seq study on human hippocampus (46) (Fig. 2F), supporting that the PSM effectively preserved the key transcriptional profiles for major brain cell types.

We further checked the major cell types contributing to the hippocampus subregion-specific markers identified at spatial spot level. In SL, the Exc was one of the major cell types expressing the subregion-specific markers due to its abundance and relatively large size in human hippocampus (Fig. 2G). The heterogeneity of the transcriptional profiles of these Exc across different subregions has contributed to the divergence of region-specific markers (**Fig. S5G**). This heterogeneity in Exc were validated at the protein level on IHC sections (**Fig. S2C-D; Fig. S3A-C**), further supporting the reliability of our approach to enhance ST data from the spatial spot level to pseudo–sc resolution. In addition, in the s.r. subregion, enriched by the synapse from Exc somas located in CA1, CA2, and CA3, *DDN* gene, a gene related to the neuron signaling transmission (55), was the marker and highly expressed by the Exc. Since Oli were highly enriched in the s.o. subregions, the Oli specific marker, *CRYAB*, was the major marker for the s.o. area. In summary, we successfully inferred the cellular composition and cell type-specific transcriptional profiles in each spot, as well as constructed a pseudo-sc matrix based on these profiles. Based on this matrix, we have revealed the heterogeneity of the Exc across subregions in SL.

### Subregion specific DGE analyses reveal the underlying mechanisms of selective vulnerability in individuals with PART and AD

**Figure 3:**
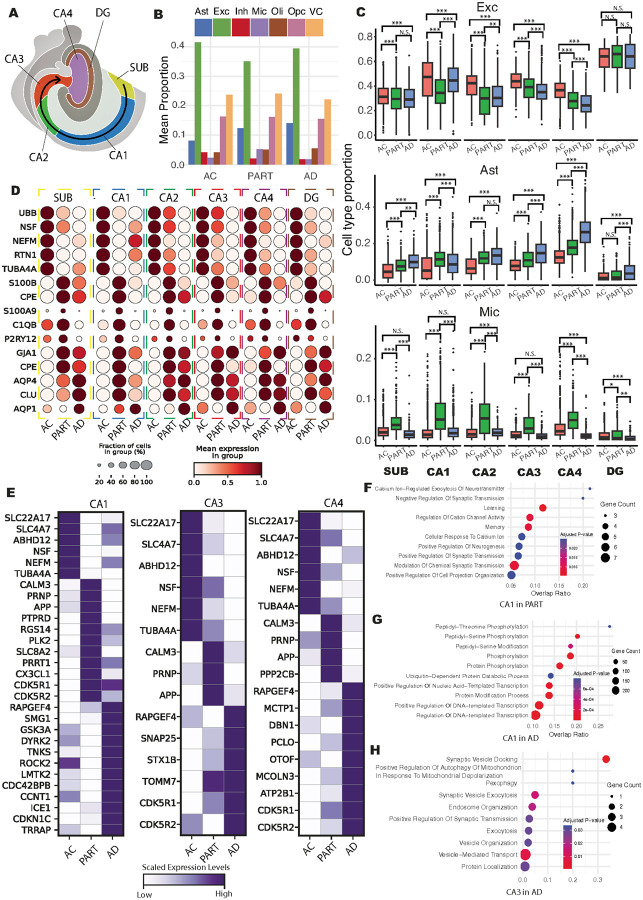
Subregion-Specific Transcriptional Divergence in the Human Hippocampus Across AC, PART, and AD (A) The anatomical structure of the SL in human hippocampus. (B) The cellular proportion of Ast, Exc, Inh, Mic, Oli, OPC, and VC in SL across the AC, PART, and AD groups. (C) Comparison of Exc, Ast, and Mic proportions across AC, PART, and AD groups within each subregion of the SL. N.S. indicates non-significant (adjusted P-value > 0.05) and * represents adjusted P-value < 0.05. ** and *** indicates adjusted P-value < 0.01 and < 0.001, respectively. (D) Significant DEGs identified from subregion-specific comparisons between AC, PART, and AD at spatial spot resolution. (E) Heatmap of the DEGs in Exc between AC, PART, and AD in CA1, CA3, and CA4 subregions. The color of the square represents the expression levels of the genes. (F-H) GO enrichment analysis of DEGs highly expressed in Exc across hippocampal subregions and disease stages. GO terms enriched in DEGs from Exc located in CA1 in the PART group compared to the AC and AD groups (F); GO terms enriched in DEGs from Exc located in CA1 in the AD group compared to the AC and PART groups (G); GO terms enriched in DEGs from Exc located in CA3 in the AD group compared to the AC and PART groups (H). The x-axis represents the proportion of enriched DEGs relative to all DEGs (overlap ratio), and the y-axis lists the enriched GO terms. Dot size indicates the number of genes associated with each GO term, and dot color reflects the adjusted *p*-value.

Selective vulnerability, a hallmark of AD, refers to the disproportionate impact of AD pathological hallmarks on neurons in specific brain regions (56). Interestingly, previous studies have also identified this hallmark in individuals with PART (57). In the hippocampus, Exc in the CA1 subregion are particularly vulnerable to developing NFTs compared to other regions (58), a pattern also observed in our samples (**Fig. S1A4-F4; Fig. S1A5-F5**). To investigate the impact of this hallmark to each specific cell types in PART and AD, we compared the region-specific cellular compositions across the AC, PART, and AD groups in the SLs (Fig. 3A). Overall, the mean proportion of Exc was highest in AC and lowest in PART (Fig. 3B). Additionally, while the proportion of Ast increased in parallel with the rising abundance of AD pathological hallmarks, Mic increased primarily in PART but decreased in AD (Fig. 3B), suggesting distinct patterns of change between these two glial cell types. The reduced proportion of Mic in AD may contribute to the increased proportion of Exc observed in AD relative to PART. In contrast to the changes observed in Exc, Ast, and Mic, the proportion of Opc, Oli, and VC maintained relatively stable across AC, PART, and AD (Fig. 3B).

To further explore regional differences in cellular composition of Exc, Ast, and Mic among AC, PART, and AD groups, we analyzed subregion-specific changes following the anatomical organization of the human hippocampus (Fig. 3A). Compared to AC group, the CA1 subregion in PART exhibited the most pronounced decrease in the proportion of Exc across all examined SLs (t-statistics=−21.04, adjusted p-value = 2.40×10^− 93^), along with the largest increases in Ast (t-statistics = 22.20, adjusted p-value = 5.01×10^− 103^) and Mic (t-statistics = 28.83, adjusted p-value = 1.18×10^− 166^) (Fig. 3C). Given that previous studies have reported limited neuronal degeneration in PART (24, 59), the observed decrease in Exc proportion in the CA1, along with increased proportions of Ast and Mic, likely reflects a decline in normal neuronal function and a pronounced glial cells reactivation against the stress. At the spatial spot resolution, compared to the AC group, the CA1 subregion exhibited the most significant downregulation of genes involved in neuronal structure, function, and signaling, including *UBB, NSF, NEFM, RTN1*, and *TUBA4A* (60). In contrast, markers of Ast (e.g., *S100B, CPE*) (61, 62) and Mic (e.g., *S100A9, C1QB, P2RY12*) (63–65) reactivation were highly enriched in the PART group (Fig. 3D). These molecular changes likely contributed to the decreased estimated proportion of Exc and increased proportions of Ast and Mic in the PART group (Fig. 3B–C). Notably, in the CA1 subregion, the divergence in the proportions of Exc, Ast, and Mic were relatively small when comparing the AC to AD groups (Fig. 3C). This pattern suggests that although glial cell activation occurs early in response to stress, it may transition into dysfunction or degeneration at the later stages of AD (66–68). The rapid decline in glial reactivation markers from the PART to the AD groups (Fig. 3D), particularly those associated with Mic (e.g., *S100A9, C1QB, P2RY12*), likely contributes to the decreased estimated proportions of these glial cell types in the CA1 region in AD, which may in turn explain the relative increase in Exc proportions in the comparison between AD and PART.

In addition to the CA1, we also found a significant reduction in the proportion of Exc in the SUB, CA2, CA3, and CA4 subregions in both the PART and AD groups compared to the AC group. Although the CA3 and CA4 exhibited less vulnerability compared to the SUB, CA1, and CA2 subregions (69), particularly in PART, the observed reduction in Exc proportions in the CA3 and CA4 suggests that these regions also experience significant stress, despite showing fewer hallmark AD pathologies. In contrast, the DG subregions showed relatively preserved Exc proportions (Fig. 3C), consistent with previous findings that granule cells, a subtype of Exc mainly located in the DG, were more resilience to the stress compared to the pyramidal Exc located in the SUB to CA4 areas (70, 71). Notably, the proportion of Exc in the CA3 and CA4 progressively declined from AC to PART and further to AD, whereas Ast proportions generally increased across most SLs over the same progression, except in CA1, where Ast proportions significantly decreased from PART to AD. This may indicate that Ast dysfunction emerges earlier in the CA1 than in other regions, which could also be reflected by the large downregulation of Ast reactivation markers in AD compared to the PART group (Fig. 3D). For Mic, most SLs displayed increased proportions in PART relative to AC, followed by a decline in AD, suggesting that microglial activation peaks in PART and diminishes at later AD stages. Since Mic are the primary phagocytes responsible for Aβ clearance (72), their dysfunction may lead to Aβ plaque accumulation observed in AD, which was also observed in our previous ST studies on prefrontal cortex from AD samples (20).

To better understand transcriptomic factors contributing to the selective vulnerability observed in PART and AD at pseudo-sc resolution, we first conducted cell type–specific DGE analyses in Exc across SLs among the AC, PART, and AD groups (**Fig. S6A; Table S4**). Given that limited pathological changes were observed in all cell types in the DG subregion (Fig. 3C), we mainly focused on the molecular divergence of SUB to CA4 subregions. Compared to the AC group, genes involved in neuronal metabolism (e.g., *SLC22A17, SLC4A7, ABHD12*) and synaptic transmission (e.g., *NSF, NEFM, TUBA4A*) were consistently downregulated in Exc across all SUB and CA subregions in both PART and AD groups (Fig. 3E; **Fig. S6B**), suggesting that despite minimal neuronal loss in PART (24, 59), functional impairments in Exc likely contribute to the observed mild cognitive decline. In PART, several genes associated with oxidative stress responses, such as *CALM3, PRNP*, and *APP* (73–76), were upregulated in Exc across the SUB to CA4 subregions compared to both AC and AD groups (Fig. 3E). Notably, Calmodulin 3 (encoded by *CALM3*) is a key subunit of phosphorylase kinase (PhK), which can phosphorylate tau and contribute to NFT formation (77). In addition, amyloid precursor protein (APP, encoded by *APP*) is the source of Aβ peptides, and its elevated expression may promote increased Aβ production and deposition, potentially facilitating progression toward AD (78). In AD, a stress response factor, *RAPGEF4*, was upregulated in Exc across the SUB to CA4 subregions compared to both AC and PART (Fig. 3E). *RAPGEF4* encodes a cAMP-regulated guanine nucleotide exchange factor involved in cAMP-mediated signaling (79), which can influence kinases that induce tau protein phosphorylation (80). Thus, the upregulation of *RAPGEF4* in AD may contribute to the exacerbation of tau pathology in the human hippocampus.

Given that all Exc in the SUB and CA subregions were under high stress but NFT accumulation was observed only in Exc within the CA1 subregion in PART, the unique Exc-specific DEGs in PART compared to AC and AD may provide insight into this selective vulnerability. When compared to the AC and AD groups, the Exc in CA1 subregion in PART exhibited the highest number of uniquely upregulated genes, in contrast to the relatively minor region-specific transcriptional changes seen in Exc in SUB and CA2-CA4 (**Fig. S6A**). Many of these CA1-specific, PART-upregulated genes in Exc were significantly enriched in GO terms related to neuroprotection and synaptic maintenance (Fig. 3F), including “Positive Regulation of Neurogenesis” (e.g., *PTPRD, RGS14*), “Positive Regulation of Synaptic Transmission” (e.g., *PLK2, SLC8A2*), and “Regulation of Neurotransmitter Receptor Activity” (e.g., *PRRT1*). These findings suggested that Exc in the CA1 subregion may activate protective and compensatory pathways in the PART group to maintain synaptic integrity and counteract neuroinflammation-induced dysfunction. We also identified *CX3CL1* as a uniquely upregulated gene in CA1 in PART (Fig. 3E). This gene encodes a neuron-derived chemokine that inhibits microglial hyperactivation, thereby reducing neuroinflammation and supporting neuronal function (81). Given the concurrent observation of heightened microglial activation in CA1 during PART, the elevated expression of *CX3CL1* likely reflects a neuronal compensatory response to suppress excessive microglial activity and limit inflammatory damage. However, since previous studies (82, 83) have shown that activated Mic are key contributors to Aβ clearance, sustained CX3CL1 upregulation may inadvertently impair microglial phagocytic function. As a result, this protective anti-inflammatory signaling could paradoxically facilitate Aβ accumulation, potentially promoting the transition from PART to AD through the formation of Aβ plaques. In contrast to genes identified in CA1, the uniquely upregulated genes in the PART group identified in the SUB and CA2–CA4 subregions did not include any genes known to directly promote NFT formation. Notably, in the PART group, *PPP1R9B* and *PPP2CB* were upregulated in relative to both AC and AD groups in SUB and CA4 regions, respectively (Fig. 3E; **Fig. S6B**). These two genes encode key regulatory components of protein phosphatase 1 and protein phosphatase 2A, respectively, both of which are primary enzymes responsible for tau dephosphorylation (84). Together, these findings highlighted transcriptomic signatures that might underlie the selective vulnerability of Exc in the CA1 subregion of the human hippocampus in PART.

Similar to PART, Exc in the CA1 region in AD exhibited the highest number of uniquely upregulated genes across all SLs when compared to both AC and PART groups (**Fig. S6A**). Notably, these uniquely upregulated genes in CA1-Exc in AD were strongly associated with NFT formation, rather than neuroprotective compensatory mechanisms observed in PART. Enriched GO terms included processes such as “Phosphorylation” (e.g., *SMG1, GSK3A, DYRK2*), “Peptidyl-Serine Phosphorylation” (e.g., *TNKS, ROCK2*), and “Peptidyl-Threonine Phosphorylation” (e.g., *LMTK2, CDC42BPB*) (Fig. 3G), highlighting molecular mechanisms that may contribute to the higher NFT burden in AD compared to PART (85). Additionally, several GO terms related to transcriptional regulation, such as “Positive Regulation of DNA-templated Transcription” (e.g., *CCNT1, ICE1*) and “Positive Regulation of Nucleic Acid-Templated Transcription” (e.g., *CDKN1C, TRRAP*), were also enriched in CA1-Exc in AD. These findings suggest that CA1-Exc in AD may be under considerable damage, potentially leading to transcriptional dysregulation and DNA damage. Importantly, such transcription-related responses were not observed in Exc populations from other SLs in AD. In the CA3 subregion, the enriched GO terms “Synaptic Vesicle Exocytosis” (e.g., *SNAP25, STX1B*) and “Positive Regulation of Autophagy of Mitochondrion in Response to Mitochondrial Depolarization” (e.g., *TOMM7*) suggest potential compensatory mechanisms for enhancing neuronal connectivity and promoting neuroprotection (Fig. 3H). Interestingly, we noticed that *CDK5R1* and *CDK5R2*, key promoters for NFT formation (86, 87), showed the highest expression levels in the AD group across all SL subregions, except in CA1, where their expression was highest in the PART group (Fig. 3E). Since NFTs were also observed in the SUB and CA2–CA4 subregions during the late stages of AD, our findings suggests that the mechanisms underlying NFT formation may be shared between PART and AD. However, the elevated expression of *CDK5R1* and *CDK5R2* in these subregions appears only at the late stage of AD, which may explain why NFTs are present in these regions in AD but not in PART.

### Glial cells resilience in PART and AD

Although predominant inflammation and Aβ production related genes were upregulated in Exc across the SUB to CA subregions in the PART group, neuronal degradation and Aβ plaque accumulation are rarely reported in PART. In addition to the intrinsic stress response mechanisms of Exc neurons, the resilience of glial cells, particularly their ability to clear excess Aβ and provide neuroprotection, also contributes to maintaining neuronal survival (46). To investigate the molecular mechanisms underlying this phenomenon, we analyzed subregion-specific transcriptional differences in glial cells across the SUB to CA4 subregions in the AC, PART, and AD groups. Since the proportions of Oli and OPC remained relatively stable across groups (Fig. 3B), our analysis primarily focused on Ast and Mic within the SLs. We observed a consistent upregulation of a stress-response gene, *NTRK2*, in Ast across all SLs in the PART group, compared to AC and AD groups (**Fig. S6C-D**). Additionally, pro-inflammatory genes such as *SERPINA3, S100B*, and *SPP1* were also elevated, suggesting that Ast was the key contributor to the inflammatory environment observed in PART (88). Similar to Exc, Ast in the CA1 region in the PART group also showed the highest number of uniquely upregulated genes among SLs (**Fig. S6A**). While no CA1-specific Ast genes were linked to NFT formation in PART, several genes associated with synaptic plasticity and neuronal survival, such as *HPCA* and *SLC44A3*, were upregulated in the PART group compared to the AC and AD groups (**Fig. S6C**), supporting the role of Ast in counteracting neurodegeneration. Furthermore, *SNX3* was uniquely upregulated in CA1 Ast in PART compared to AC and AD. Sorting nexin 3, encoded by *SNX3*, has been shown to inhibit Aβ generation by altering APP trafficking, and may underlie the absence of Aβ plaque deposition in the CA1 region in PART. Compared to CA1, the number of uniquely upregulated genes in Ast in PART versus AC and AD was lower within the SUB and CA2–CA4 subregions (**Fig. S6A**). Furthermore, within these genes, only a few of them, such as *S100A13* in SUB as well as *GLUL* and *UQCRB* in CA4 (**Fig. S6D**), have been reported to be associated with neuroinflammation and AD pathological hallmark (89, 90). This pattern indicates a lower level of Ast reactivation in these regions compared to CA1, suggesting less Exc damage.

Notably, in the AD group, the gene, *SPARC*, which affects two central pathological features of AD: Aβ deposition (91) and blood-brain barrier (BBB) disruption (92), is among the common genes upregulated in AD compared to AC and PART across SLs (**Fig. S6C-D**). In the CA1 subregion, unlike Ast in PART, which primarily upregulated pro-inflammatory genes, Ast in AD display a dual phenotype. On one hand, they express genes associated with axonal and synaptic degradation (e.g., *DPYSL2, RGMA, GABBR1, TRIM2*) (**Fig. S6C**). On the other hand, they also upregulate genes related to compensatory neuroprotective mechanisms, such as antioxidative stress responses (e.g., *GPX4, SELENOW*), metabolic support (e.g., *UGP2, PFKP, PRDM16*), and inflammation resolution (e.g., *ZFP36L2*). This dual profile aligns with previous studies showing that Ast exert both neuroprotective and neurotoxic effects in the later stages of AD (93). Similar patterns were also observed in Ast within the CA3 and CA4 subregions in AD, whereas such patterns were not evident in the SUB and CA2 subregions. Further, we have also identified several unique upregulated genes in Ast in the AD group that are related to the BBB disruption, including *TGFB2* in CA3 (94) and *PTK2B* in CA4 (95) (**Fig. S6D**). Together, the upregulated transcript molecules in Ast in AD, compared to AC and PART, exhibited both neuroprotective functions and, paradoxically, contributed to neurotoxin as well as blood-brain barrier disruption.

Due to the limited numbers and relatively small size of Mic, fewer Mic-specific DEGs were detected across the SUB and CA subregions in the AC, PART, and AD groups (**Fig. S6A, Table S4**). Similar to Ast, Mic showed prominent reactivation across the subregions, evidenced by increased SPP1 expression in the PART group compared to both AC and AD groups. In the CA1 subregion, Mic from PART samples showed upregulated expression of autophagy-related genes (e.g., *GABARAP, OTUB1, UCHL1*) relative to those from AC and AD samples, suggesting enhanced Aβ clearance capacity (**Fig. S6E**). In CA4, Mic in PART exhibited even stronger expression of genes directly linked to both Aβ clearance and autophagy (e.g., *APOC1, HNRNPC*), indicating a robust neuroprotective response in this subregion (**Fig. S6E**). This may help explain the relative resistance of CA4 to Aβ aggregation compared to CA1, consistent with prior findings (26, 96). In contrast, Mic from SUB, CA1, and CA4 regions showed limited upregulation of Aβ clearance genes in AD samples compared to AC and PART. Instead, their transcriptomic profiles were enriched for genes associated with pro-inflammatory responses. Combined with the marked reduction in Mic proportions from PART to AD across SUB and CA subregions, these findings suggested a progressive decline in Aβ clearance capacity, contributing to plaque accumulation in AD. Moreover, dysfunctional Mic likely exacerbate neuroinflammation, further damaging Exc.

### Subregion communications within human hippocampus in AC, PART, and AD

**Figure 4:**
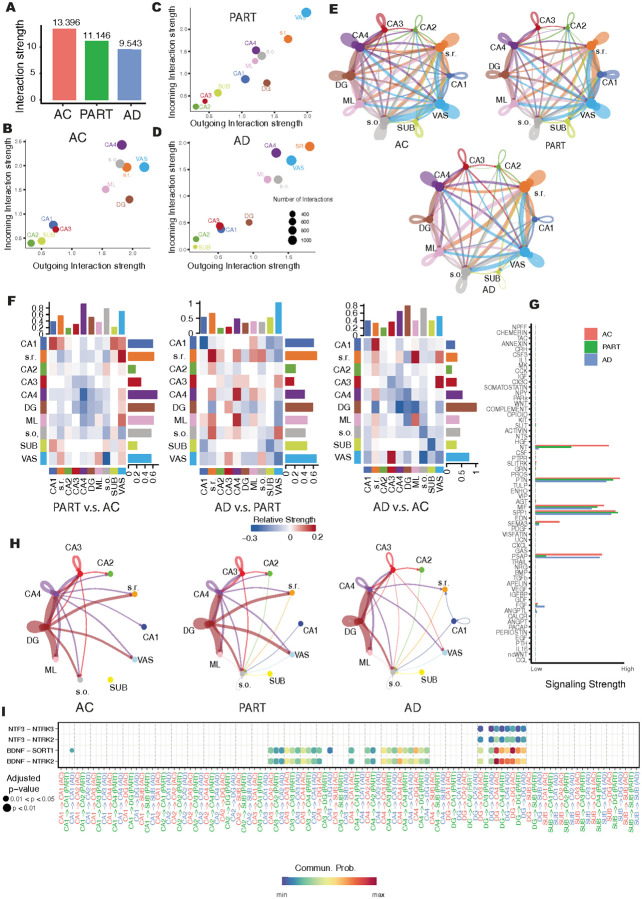
Inter-subregion interactions among human hippocampus in AC, PART, and AD (A) Interaction strength of the subregion-subregion interactions in AC, PART, and AD, respectively. (B-D) Outgoing and incoming interaction strength of hippocampal subregions in AC (B), PART (C), and AD (D) groups. The x-axis indicates the strength of outgoing interactions, while the y-axis indicates the strength of incoming interactions for each subregion. Dot color represents the subregion, and dot size corresponds to the total number of outgoing and incoming interactions combined. (E) Subregion-subregion communication networks in the AC, PART, and AD groups. Each dot represents a hippocampal subregion, colored according to its identity. Edges represent inferred inter-subregion interactions, with line color indicating the source subregion and line width corresponding to the interaction strength. (F) Pairwise comparison of subregion-subregion communication networks across AC, PART, and AD groups. The x-axis represents the strength of outgoing interactions, and the y-axis represents the strength of incoming interactions for each subregion. Bar plots show the total interaction strength, with bars oriented along the x-axis for outgoing interactions and along the y-axis for incoming interactions. (G) Signaling pathway strength across subregion-to-subregion interactions in AC, PART, and AD groups. The x-axis is the signaling strength, and y-axis indicates the signaling pathways. The color represents AC, PART, and AD groups. (H) The subregion-subregion interaction networks involved in the NT signaling pathway. Each dot represents a hippocampal subregion, colored according to its identity. Edges represent inferred inter-subregion interactions, with line color indicating the source subregion and line width corresponding to the interaction strength. (I) The communication strength comparison of the specific LR pairs of NT signaling pathways across AC, PART and AD groups. The x-axis represents the direction of the LR pairs, and the y-axis indicates the specific LR pairs. Permutation test and Bonferroni correction were used for adjusted *P*-value calculation. Dot size represents the adjusted p-value, and the color reflects the communication strength of the LR pairs.

The interactions between subregions of the human hippocampus are essential for coordinating memory encoding, consolidation, and retrieval across various cognitive contexts (97, 98). As a result, communication between hippocampal subregions may be alternated or disrupted, contributing to the cognitive decline seen in PART and, more prominently, in AD. To investigate how subregional communication patterns change with increasing abundance of AD pathological hallmarks, we first constructed interaction networks among all hippocampal subregions at spatial spot levels in individuals with AC, PART, and AD, respectively. Given that interaction strength between subregions may decrease with increasing Euclidean distance (99), we incorporated spatial distance as a covariate in the construction of the interaction networks. In general, the strength of the communication among all subregions was decreasing from AC to AD (Fig. 4A). While the VAS and s.r. subregions exhibit relatively high incoming and outgoing interactions across the AC, PART, and AD groups, the SUB, CA2, and CA3 subregions display comparatively lower interaction strengths among these groups (Fig. 4B–D). Specifically, SUB and CA1 exhibited a significant increase in both outgoing and incoming signaling strength in PART compared to AC, followed by a marked decrease in AD relative to PART. In contrast, CA3 and CA4 showed reduced signaling strength in PART compared to AC, but this increased in AD relative to PART (**Fig. S7A**). Besides, the VAS showed the highest strength of the incoming signaling in PART compared to AC and AD, but exhibited a gradually decreased strength of the outgoing signaling with the increase of the AD pathological hallmarks. These findings suggested a dynamic, subregion-specific reorganization of hippocampal communication in response to PART and AD pathology.

To pinpoint the divergence of subregion-specific communication patterns across the AC, PART, and AD groups, we first analyzed the interaction strength between all pairs of hippocampal subregions. The communication networks among hippocampal subregions varied across the AC, PART, and AD groups (Fig. 4E). The pairwise comparison of interactions strengths among subregions illustrated that the interaction strength among multiple SLs decreased in the PART group compared to the AC group, followed by an increase in the AD group relative to PART. However, overall strength of these interactions in AD remained lower than in AC (Fig. 4F). The divergence of these interaction strength between AC, PART, and AD was primarily driven by changes in signaling pathways involving neurotensin (NT), pleiotrophin (PTN), macrophage migration inhibitory factor (MIF), SPP1, semaphorin 3A (SEMA3A), and prosaposin (PSAP) (Fig. 4G). Among these interactions with divergence strength, the NT signaling exhibited the greatest progressive decline from AC through PART to AD. Previous studies suggested that the NT signaling pathway plays a critical role in chemical neurotransmission, involving a variety of neurotransmitters that act on diverse receptors, often through co-release and feedback mechanisms that fine-tune neuronal responses (100). Additionally, NT can both promote neuronal survival and induce neuronal apoptosis, depending on the cellular context and receptor signaling pathways involved (100, 101). Thus, the pathological disruption of NT signaling observed in our data may contribute to the neuron damage in PART and AD. In the AC group, NT signaling primarily originated from the CA3 and CA4 regions, projecting to other SLs such as CA1 and CA2, respectively. Additionally, NT signaling from the DG was mainly directed toward CA3 and CA4, as well as glial cell-enriched subregions including the s.r., s.o., and ML (Fig. 4H). This pattern aligned with previous findings showing that granule cells in the DG form strong connections with Exc in CA3 and CA4, but have limited direct connectivity with CA1 and CA2 (102). However, in the PART and AD groups, the NT signaling from CA4 to CA1 and CA2 was dramatically reduced compared to the AC group, likely due to pathological changes in the gene expression patterns of the ligands and receptors in cells located in these regions. Interestingly, in the AD group, cells in the CA1 subregion exhibited upregulated intra-region ligand-receptor (LR) pairs involved in NT signaling (Fig. 4H), likely reflecting an effort to preserve signaling activity within CA1 under the severe stress induced by AD pathology.

To illustrate the impact of AD-related pathological hallmarks on NT signaling, we analyzed the pathological divergence of specific LR pairs involved in this process (Fig. 4I). Notably, the neuroprotective LR pair BDNF–NTRK2, originating from CA3 and projecting to CA2 and CA4 subregions, showed a progressive decline in interaction from AC to PART, and further to AD. A similar decreasing trend was observed from DG to CA4 and ML subregions. Given that BDNF binding to NTRK2 activates key intracellular signaling pathways, including PI3K–AKT and MAPK–ERK, which are essential for neuronal survival and resilience to cellular stress (103), the reduced interaction of this LR pair likely reflects a loss of inter-subregion neurotrophic support, driven by increasing pathological burden in AD. In addition, we have noticed one cell apoptotic related LR pairs, BDNF-SORT1, was decreased among the CA2, CA3, CA4, and DG in the AD group compared to the AC and PART groups. SORT1 acts as a co-receptor for neurotrophins such as BDNF, and when bound to BDNF, it can trigger neuronal apoptosis (104). The reduced interactions of BDNF–SORT1 across several hippocampal regions may reflect a compensatory mechanism aimed at preserving neuronal integrity. In contrast, we found that BDNF–SORT1 interactions increased specifically in the CA1 subregion in the AD group compared to the AC and PART groups. Given that Exc in CA1 are among the most vulnerable to degeneration in AD, this heightened interaction may contribute to the severe neuron death in CA1.

In addition to NT, we have observed significant variations in interaction strength within the PTN signaling pathway, which exhibited the strongest interactions strength among all signaling pathways across the hippocampal subregions (Fig. 4G). Previous studies have proposed that PTN promotes hippocampal neurogenesis by stimulating neural progenitor proliferation through the activation of AKT signaling (105) and stabilizes dendritic microtubules of the damaged neurons (106). Furthermore, PTN also acts as a protector of the BBB by supporting pericyte function, promoting angiogenesis, and facilitating vascular repair (107). In our data, we have observed that the PTN signaling pathway from multiple regions, including SUB to CA3, to VAS, was disrupted (**Fig. S7B**). In addition, among the interactions involved in the PTN, the PTN-(ITGAV + ITGB3) LR pair, which directly supports the BBB integrity, was detected from almost all subregions in hippocampus to the VAS in the AC and PART group, but diminished in the AD group (**Fig. S7C**). Given that PTN binding the ITGAV and ITGB3 can enhance the adhesion of endothelial cells located on the surface of the large blood vessels and thus maintain the BBB structural stability and prevent leakage (108), the disruption of this interaction may contribute to the BBB disruption observed in late stage of AD.

In summary, our inter-subregion analysis identified specific molecular mechanisms supporting neuronal survival and BBB maintenance that are disrupted in AD but preserved in PART, offering insights into the neuronal degeneration and BBB breakdown seen in late-stage AD but not in PART.

### Cellular interactions between VAS and the nearby cells

BBB disruption is a hallmark of late-stage AD (109), primarily driven by the activation of astrocytes and microglia in response to heightened cellular stress (68). This breakdown permits neurotoxic substances to infiltrate brain tissue (109), with their accumulation often occurring around large blood vessels. The resulting localized damage can propagate to nearby regions, contributing to further pathological changes in adjacent small vessels (110). Further, these pathological changes in adjacent small vessels can significantly influence the behavior of the glial cells, especially Ast and Mic, which are implicated in propagating neuroinflammation, disrupting synaptic support, and facilitating neuronal apoptosis (111, 112). As a result, the cells in close proximity to the large vessels form a distinct microenvironment compared to those in distal regions, and uncovering the differences in cellular interactions between areas near and far from large vessels may be critical for understanding the mechanisms of vessel-induced neuronal degeneration.

**Figure 5:**
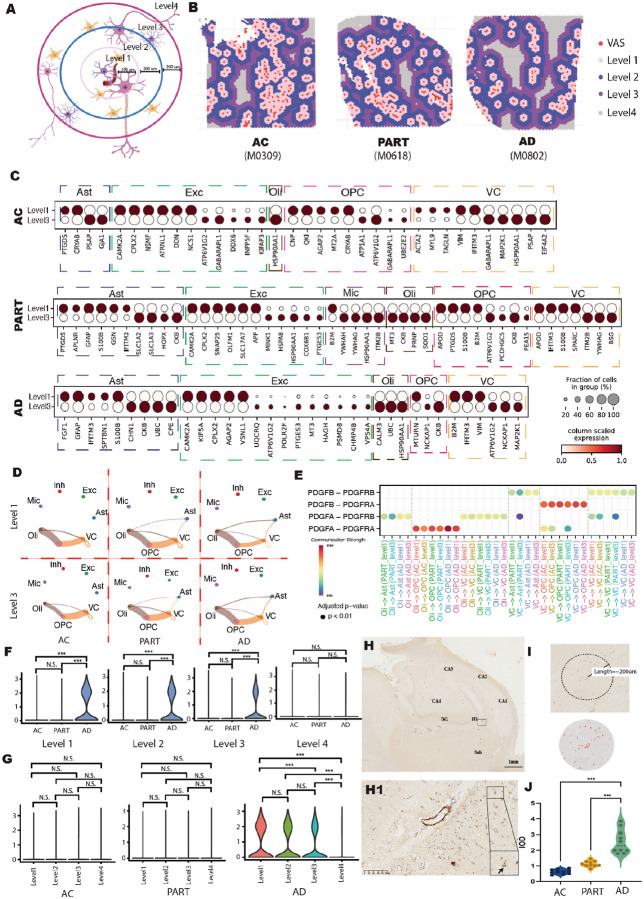
Transcriptional divergence between cells located near and distant from large blood vessels (A) Concentric analysis. (B) The location of the VAS and the cells located in level1 to 4. (C) The DEGs of the cells between level1 and 3 in the AC, PART, and AD groups, respectively. Dot size represents the proportion of cells expressing each marker, while color intensity indicates the expression level of the corresponding marker. (D) The cell-cell interaction networks involved in PDGF signaling pathway. Each dot represents a hippocampal subregion, colored according to its identity. Edges represent inferred inter-subregion interactions, with line color indicating the source subregion and line width corresponding to the interaction strength. (E) The communication strength comparison of the specific LR pairs of PDGF signaling pathways across AC, PART and AD groups. The x-axis represents the direction of the LR pairs, and the y-axis indicates the specific LR pairs. Permutation test and Bonferroni correction were used for adjusted P-value calculation. Dot size represents the adjusted p-value, and the color reflects the communication strength of the LR pairs. (F) Comparison of the expression levels of *TP53INP2* in Ast between AC, PART, and AD groups in level1 to 4, respectively. N.S. indicates non-significant (adjusted *P*-value > 0.05) and *** represents adjusted P-value < 0.001. (G) Comparison of the expression levels of *TP53INP2* in Ast between level1 to level4 in AC, PART, and AD groups, respectively. N.S. indicates non-significant (adjusted P-value > 0.05) and *** represents adjusted P-value < 0.001. (H) Experimental validation of the expression of TP53INP2 in Ast in AD samples through IHC. (I) Selection of vessel-adjacent regions for spatial analysis. The upper panel shows a representative large blood vessel with a surrounding 200 μm radius (dashed circle) indicating the defined perivascular region. The lower panel highlights the area for downstream analysis. (J) Comparison of the integrated optical density (IOD) of TP53INP2 in the selected area between AC, PART, and AD.

To address this, we first identified the location of each VAS spot and conducted a concentric analysis using a strategy similar to our previous study (52). Spatial spots were categorized into four levels (1 to 4) based on their distance from the VAS (Fig. 5A–B). Due to the proximity and minimal transcriptional divergence between levels 1 and 2, our DEG analysis focused on comparing level 1 (proximal) and level 3 (distal) spots. We found that while most DEGs identified in AC and PART groups were functionally similar, primarily related to neuronal maintenance, *APP* was significantly upregulated in Exc in level 1 compared to level 3 spots specifically in the PART group, but not in the AC or AD groups (Fig. 5C). This suggested that Exc near large vessels in PART may produce excessive APP, potentially enhancing Aβ plaque formation relative to distal neurons. In contrast, the absence of *APP* upregulation in the AD group may indicate that Exc near large vessels undergo apoptosis due to severe stress, leading to a reduction in *APP* expression in these neurons (113). Additionally, the inflammation-related gene *S100B* was upregulated in Ast in level 1 compared to level 3 in both PART and AD, but not in AC, indicating a stronger inflammatory response in vessel-proximal Ast within these conditions. In the AD group, downregulated genes in Exc in level 1 spots were associated with responses to mitochondrial dysfunction (e.g., *UQCRQ, ATP6V1G2, POLR2F*), oxidative stress (e.g., *PTGES3, MT3, HAGH*), and protein clearance (e.g., *PSMD8, CHMP4B, VPS4A*), suggesting greater functional impairment in Exc near large vessels in AD (Fig. 5C). Conversely, the upregulation of the energy metabolism-related gene *KIF5A* (114) in level 1 spots in AD may reflect a compensatory response to mitochondrial stress in these neurons (Fig. 5C). Together, these findings highlight significant cell type-specific transcriptional differences in vessel-adjacent regions across AC, PART, and AD groups, potentially shedding light on the detrimental impact of BBB disruption in AD.

We next constructed cell–cell communication networks within each level for the AC, PART, and AD groups to identify potential cellular interactions involved in neuroinflammation and degeneration (**Fig. S8A**). Two key cellular survival-related signaling pathways, GAS and PDGF, originating from VC and targeting multiple cell types, particularly Ast, Mic, and Oli, were detected in levels 1 and 3 spots across the different groups (**Fig. S8B-C**; Fig. 5D). For the GAS signaling pathway, interactions were predominantly directed from VC to Mic in both the AC and PART groups, but were prominent from VC to Ast in the AD group (**Fig. S8C**). Specifically, GAS6–MERTK interactions from VC to Mic were absent in the AC group and progressively increased in PART and AD, while GAS6–MERTK interactions between VC and Ast were only detected in the AD group This result is consistent with previous studies showing that the GAS6–MERTK pair plays a dual role in AD: while it promotes Aβ clearance via microglial activation, it can also drive neuroinflammation through Ast reactivation (115, 116). Similar to GAS signaling, PDGF signaling from VC to Ast was observed only in the PART and AD groups, but not in AC (Fig. 5D). Specifically, PDGFB–PDGFRB interactions showed a progressive increase from AC to PART to AD (Fig. 5E). This LR pair was similarly strong in both levels 1 and 3 in the AD group, while it was stronger in level 1 than in level 3 in the PART group. PDGFB–PDGFRB signaling from VC to Ast activates Ast to recruit monocytes into the brain, thereby amplifying neuroinflammation and leading to neuron apoptosis (117). In summary, our results indicated that the pathological alterations in the transcriptional profiles of Ast located near large vessels was activated by VC in AD, which may play a critical role in accelerating disease progression.

To further investigate transcriptional alterations in Ast located near large vessels during disease progression, we performed DGE analysis in Ast between the AC, PART, and AD groups across levels 1 to 4, respectively. Notably, the P53-related gene *TP53INP2* was significantly upregulated in Ast in the AD group compared to both AC and PART groups at levels 1 to 3 (Fig. 5F). However, this upregulation was not observed at level 4. This spatial pattern suggests that TP53INP2 is highly enriched in Ast located near large vessels in AD. *TP53INP2* (Tumor Protein P53 Inducible Nuclear Protein 2) encodes a stress-responsive nuclear protein known to promote cellular autophagy (118). Excessive expression of TP53INP2 may lead to hyperactivation of autophagy in Ast, contributing to remove misfolded proteins and toxic materials of nearby Exc to maintain the neuron health (119). Meanwhile, as a tumor suppressor, TP53INP2 also promote cellular apoptosis under pathological conditions (120), which may further exacerbate Exc degeneration. To validate the upregulation of TP53INP2 observed in our ST data, we performed IHC staining on six hippocampal samples used for ST and one hippocampal sample used for snRNA-seq, and included one additional independent hippocampal samples to the AC and PART groups, respectively. (**Table S1**; Methods). TP53INP2 protein was enriched around large vessels in all groups, with significantly elevated expression in astrocytes near vessels from AC to PART to AD, consistent with our ST data (Fig. 5H–J; **Fig. S8D-E**). Together, these results suggest that BBB disruption in large vessels leads to pathological alterations in the transcriptional profiles of nearby cells. In particular, the elevated expression of *TP53INP2* in Ast adjacent to large vessels may play a central role in the neurodegenerative processes observed in AD.

## DISCUSSION

In this study, we generated a comprehensive, data-driven, transcriptome-wide molecular atlas of the adult human hippocampus using the advanced 10x Visium ST platform to investigate molecular alterations across AC, PART, and AD. By integrating cutting-edge ST with innovative analytical strategies, we unveiled potential mechanisms driving the transition from PART to AD and offer novel insights into AD pathology. Furthermore, our analysis pipeline, enhancing spot-level data to pseudo-sc resolution, can be applied to existing non–sc ST datasets from other brain region (18, 121), enabling deeper exploration of molecular mechanisms underlying AD.

Here, we highlight several key findings. Although multiple earlier ST studies have mapped hippocampal subregions in humans (26, 38, 39), none have successfully identified and validated markers that distinguish the CA1 and CA2 regions. In our study, we identified NRIP3 as a canonical marker that reliably delineates the boundary between CA1 and CA2. This marker provides a robust criterion for distinguishing these two subregions and offers a valuable tool for investigating region-specific pathological changes in AD. To address the relatively low resolution of the 10X Visium platform, we applied BayesPrism (45), a robust deconvolution method, to enhance spatial resolution beyond the spot level and achieve pseudo-sc resolution. At this higher resolution, we found that the distinct subregional markers of Exc within SLs, initially identified at the spatial spot level, were largely driven by transcriptional heterogeneity within the Exc population. Additionally, pseudo-sc analysis revealed changes in both cellular composition and cell type–specific transcriptional profiles across hippocampal subregions between the AC, PART, and AD groups. The observed shifts in cell proportions and gene expression suggest that PART may represent a transitional state between AC and AD, consistent with findings by Duyckaerts *et al*. (122). In the PART group, Exc in the CA1 region with NFT burden showed expected signs of stress. Interestingly, Exc in other SL regions without NFTs also exhibited elevated stress markers, likely due to age-related oxidative stress and neuronal inflammation. Notably, while Stein-O’Brien et al. (25) reported upregulation of *APP* expression in NFT-bearing Exc, we observed increased APP expression even in Exc without NFTs, particularly in the SUB and CA2–CA4 regions of the PART group. This suggests that APP upregulation may result from stress independent of NFT pathology, potentially promoting Aβ production. Given that excess Aβ is cleared by Mic, the increased proportion of Mic in PART compared to the AC group may reflect a compensatory response aimed at Aβ clearance. However, prolonged Mic activation and exposure to Aβ can lead to Mic dysfunction and degeneration (123), leading to Aβ accumulation and plaque formation. The resulting Aβ deposition intensifies inflammation, which in turn exacerbates tau phosphorylation, increasing NFT burden and compromising BBB integrity. This feed-forward loop accelerates the transition from PART to AD. Ultimately, the combined effects of Aβ plaques, NFTs, and BBB disruption lead to Exc degeneration.

Furthermore, our study identified molecular mechanisms underlying the selective vulnerability of Exc in the CA1 subregion in both PART and AD. In PART, although Exc across SUB and CA areas experienced oxidative stress and inflammation, Exc in CA1 uniquely upregulated NFT-associated genes *CDK5R1* and *CDK5R2* compared to both the AC and AD groups. In contrast, these genes were significantly enriched in the AD group, relative to AC and PART, in the SUB and CA2–CA4 subregions. Additionally, in PART, the region-specific upregulation of tau dephosphorylation-related genes, including *PPP1R9B* in SUB and *PPP2CB* in CA4, may contribute to the lower NFT burden observed in these regions. Beyond neuronal changes, we also observed enhanced glial resilience in PART, particularly in CA1, where Ast and Mic were robustly reactivated, potentially contributing to neuroprotection. However, in AD, this glial reactivation, especially in Ast, adopted a dual role: while still supporting neuronal survival, it also promoted BBB disruption, thereby exacerbating disease progression.

Analysis of inter-subregion communication in the hippocampus revealed a progressive decline in both neuronal survival and BBB integrity from AC to PART and, ultimately, to AD. BBB disruption was associated with altered transcriptional profiles in VC, Ast, and Mic near large vessels, leading to reshaped cellular interactions. Specifically, VC engaged in pro-inflammatory signalling with glial cells, particularly Ast, thereby exacerbating neuroinflammation and promoting cell apoptosis. Notably, we observed a significant upregulation of *TP53INP2* in Ast located near large vessels in the AD group, compared to the AC and PART groups. While this gene has not been widely reported in AD, a closely related gene, *TP53INP1*, has been implicated in AD pathogenesis in several studies (138, 139). As a tumor suppressor, *TP53INP2* may participate in a previously unrecognized Ast-related neural apoptosis pathway, potentially contributing to neurodegeneration. This finding warrants further validation through molecular and functional studies.

Despite the significant advances and insights provided by this study, several limitations should be acknowledged. First, although all six samples were age-matched (ranging from 79 to 92 years), the sex distribution was unbalanced (four males and two females), potentially introducing sex-specific bias. Second, the sc reference dataset used for deconvolution was not derived from the same individuals as the ST samples, which limited our choice of deconvolution methods and may have introduced analytical bias. Additionally, while we enhanced spatial resolution from spot-level to pseudo–single-cell resolution using deconvolution algorithms, the resulting pseudo-sc matrix is still computationally inferred. As such, discrepancies may exist compared to true sc resolution platforms, such as Visium HD or Stereo-seq (53). Nevertheless, despite these limitations, our study provides valuable novel insights into the transcriptional landscape of PART and AD in the human hippocampus, offering a systematic and high-resolution view of disease progression and the molecular mechanisms driving neurodegeneration.

## METHODS

### Study subjects

This study was approved by the Ethics Committee of Xiangya School of Medicine in Central South University (2020KT-37, 4/10/2020; #2023-KT084, 6/21/2023), and conducted in accordance with the Code of Ethics of the World Medical Association (Declaration of Helsinki). Brains were banked through a willed body donation program, with donors’ clinical records collected when available (124). The brains were assessed for neuropathological changes following the Standard Brain Banking Protocol established by the China Brain Bank Consortium (125). Six postmortem human hippocampal samples from Chinese Han individuals aged 79–95 years were analyzed using ST and were classified into AC, PART, and AD based on 6E10 (BioLegend, #SIG-39320) IHC for Aβ plaques (**Fig. S1A3-F3**) as well as AT8 (Invitrogen, #MN1020) and Gallyas staining (126) for NFTs (**Fig. S1A4-F4; Fig. S1A5-F5**). Specifically, the brains in the AC group were absent of Aβ deposition (Thal phase 0) and contained a few hyperphosphorylated tau (pTau) positive neurons (pre-tangle) but no Gallyas stained mature or ghost tangles in the hippocampal formation (Braak stage I-II). The two brains in the PART group showed only a few diffusion plaques in the prefrontal cortex (Thal phase 0), with AT8 and Gallyas stained NFT observed in the hippocampus and temporal lobe cortex (Braak stage III). The two brains in the AD had high Aβ plaque burden (Thal phase III) and heavy NFT pathology (Braak stages IV–V). In addition to the two AD cases included in the ST analysis, hippocampal tissue from the third AD individual was included for snRNA-seq. For the TP53INP2 IHC validation, we have added one additional sample to both the AC and PART groups. For details on human samples used in this study, please see Supplementary Table 1.

### Tissue preparation, FFPE section, 10X Visium library preparation, sequencing, data preprocessing, and IHC

Human hippocampal samples were fixed in 4% paraformaldehyde solution (Cat # G1101–500ML, Servicebio) for 24–48 hours at 4°C to preserve morphology and RNA integrity. Fixed tissues were processed using standard dehydration and paraffin-embedding protocols (127). FFPE blocks were sectioned at 5 μm thickness using a rotary microtome and mounted onto Visium Spatial Gene Expression Slides (10X Genomics), ensuring optimal placement over the capture area. Sections were baked at 60°C for 30 minutes, followed by deparaffinization, H&E staining, and imaging using a brightfield microscope to guide tissue annotation. Spatial gene expression profiling was performed using the Visium Spatial Gene Expression for FFPE protocol (10X Genomics), including probe hybridization, ligation, and amplification steps to construct spatially barcoded libraries. Sequencing was carried out on an Illumina NovaSeq 6000 platform with paired end reads, targeting a depth of at least 25,000 reads per capture spot. Raw sequencing data were processed using the 10X Genomics Space Ranger pipeline to align reads and generate spatial gene expression matrices for downstream analysis.

Raw sequencing data (BCL files) were first demultiplexed using *cellranger mkfastq* (10X Genomics), generating FASTQ files for downstream analysis. Quality control on FASTQ files was performed using *FastQC* to assess read quality, adapter content, and duplication rates. The FASTQ files were then processed with SpaceRanger (v2.1, 10X Genomics), aligning reads to the GRCh38 human genome panel to generate the count matrix.

Nuclei were isolated from frozen post-mortem brain tissue following a published protocol (128) for snRNA-seq (10X Genomics). In brief, approximately 40 mg of frozen, pulverized tissue was homogenized in chilled Nuclei EZ Lysis Buffer (MilliporeSigma #NUC101) using a glass dounce with about 15 strokes per pestle. The homogenate was passed through a 70 μm mesh strainer and centrifuged at 500 × g for 5 minutes at 4°C. The pellet was resuspended in EZ Lysis Buffer, centrifuged again, and then transferred into nuclei wash/resuspension buffer (1x PBS, 1% BSA, 0.2 U/μL RNase Inhibitor). Nuclei were washed and centrifuged three times in this buffer before being stained with DAPI (10 μg/mL). For snRNA-seq, libraries were prepared using the Chromium Single Cell 3’ Reagent Kits v3 according to the manufacturer’s protocol (10x Genomics). Sequencing was carried out on an Illumina NovaSeq 6000 platform with paired end reads, targeting a depth of at least 20,000 reads per capture nucleus. Raw scRNA-seq data were processed through cell ranger (10x Genomics) to be converted into the gene expression matrix.

For the IHC validation, FFPE blocks were sectioned at 5 μm thickness and mounted onto slides. The sections were placed under a vented hood for air drying prior to FIBCD1, NRIP3, CCK, NEFM, STXBP6, and TP53INP2 staining. FIBCD1 (Cat # 25125–1AP, Proteintech), NRIP3 (Cat # 15664–1-AP, Proteintech), CCK (Cat # 13074–2-AP, Proteintech), NEFM (Cat # 25805–1-AP, Proteintech), STXBP6 (Cat # 10976–4-AP, Proteintech), TP53INP2 (Cat # PA5–72961, ThermoFisher) were used for IHC staining according to the vendor’s instructions.

### Bioinformatics analysis of ST data of human hippocampus

ST data integration and clustering analysis for 10X Visium spatial spots At the spot level, we applied PRECAST (37), a data integration and unsupervised clustering method for ST data across multiple tissue slides, to identify subregions within the human hippocampus. PRECAST uses a two-layer hierarchical model comprising an integration layer and a clustering layer. In the integration layer, spatial spots from all tissue sections were projected into a shared low-dimensional embedding space. To preserve spatial continuity in gene expression patterns, an intrinsic Conditional Autoregressive (CAR) model was applied, encouraging nearby spots to have similar embeddings. Following integration and dimension reduction, spatial spots were clustered based on both their low-dimensional embeddings and physical coordinates. We selected K = 15 as the optimal number of clusters, guided by the elbow point of the Bayesian Information Criterion (BIC) curve. Each resulting cluster was annotated using specific gene markers. To validate our annotations, we visualized the spatial distribution of annotated spots for each sample based on their coordinates and aligned them with corresponding Eosin-stained sections. Sample orientation was verified by identifying the directions of the SUB, CA, and DG subregions on both ST data and Eosin-stained image.

#### DGE analysis

The “sc.tl.rank_genes_groups” function from the Scanpy package (v1.9.3) was utilized for DGE analysis. The Wilcoxon signed-rank test with FDR adjustment was applied to calculate the adjusted P-values, and the genes with adjusted P-value < 0.05 were considered as the DEGs.

#### Deconvolution analysis

In our study, we applied the BayesPrism algorithm (45) to enhance spatial resolution beyond the spatial spot level. Using snRNA-seq data on the human hippocampus, consisting of our in-house and public available data, we first inferred the proportion of each cell type within each spatial spot and subsequently estimated cell type-specific gene expression patterns for each spot. Briefly, BayesPrism employs a Bayesian framework that models prior distributions based on scRNA-seq data and infers the joint posterior distribution of cell type porportions and gene expression, conditioned on each spatial spot. To prepare the input data, we performed QC by filtering out outlier genes in the snRNA-seq dataset using the “*plot.bulk.outlier*” function with default parameters. We then combined the snRNA-seq and ST data into a BayesPrism object using the “new.prism” function, followed by running the “run.prism” function to estimate cell type proportions and infer gene expression profiles for each cell type in each spot.

To assess the accuracy of BayesPrism’s deconvolution performance, we compared it with three additional spatial deconvolution algorithms: CARD (49), SpaCET (50), and PANDA (51). For CARD, we used the “CARDObject” function to construct the input object and applied “CARD_deconvolution” to estimate cell type proportions per spatial spot. For SpaCET, we used “create.SpaCET.object.10X” and “SpaCET.deconvolution”, both with default parameters. For PANDA, we employed the “sc_train” and “st_train” functions to estimate both cell type proportions and gene expression profiles per spot. To evaluate consistency between BayesPrism and the other methods, we calculated pairwise Spearman correlation coefficients between the cell type proportions inferred by BayesPrism and those estimated by CARD, SpaCET, and PANDA.

#### Inter-subregion and cell-cell communication analysis

Inter-subregion and cell-cell communication analyses were performed using CellChat (v2.1.0) (129) based on the expression of known LR pairs across different subregions and cell types. For inter-subregion and cell-cell communication, we computed communication strength by modeling ligand-receptor interactions between spatial spots labeled by subregion in AC, PART, and AD samples separately. This modeling was based on the Law of Mass Action and incorporated gene expression profiles projected onto a protein-protein interaction network. Additionally, subregion/cell proportion was incorporated to minimize bias arising from unequal comparisons of inter-subregion/cell-cell interactions. To include spatial context in inter-subregion communication, we used the spatial coordinates of each spot as a cofactor. In contrast, for constructing cell-cell communication networks, we did not include spatial location as a covariate due to the limited spatial distance variation among spots within each level. We followed the official workflow and applied the data processing functions “identify OverExpressedGenes” and “identifyOverExpressedInteractions”. The inter-subregion communication networks were inferred by the function “computeCommuProb”. Function “netVisual_bubble” was used to compare the communication probabilities mediated by L-R from certain subregion group to other groups. All the analysis were performed with the default parameter setting.

#### Concentric circle analysis

To understand how gene expression varies with proximity to the large vessels with BBB damaged, we mapped the VAS on a two-dimensional panel based on their coordinator for each sample, respectively, and drew three concentric circles around each high stress focal point to differentiate spatial spot distances. We first selected spatial spot located within 600 pixels (~ 600 um) from the VAS in each sample. Spots within a radius within a radius of 200 units of the pixel (~ 200 um) are categorized as level I, those between 200 and 400 units (approximately 200–400 um) as level II, and those in 400 to 600 units (approximately 400 to 600 um) as level III. Spots intersecting circles from multiple VAS areas were assigned to the closest level.

## Supplementary Material

Supplementary Files

This is a list of supplementary files associated with this preprint. Click to download.

- SupplementalTable1final.xlsx
- SupplementalTable2final.xlsx
- SupplementalTable3final.xlsx
- SupplementalTable4final.xlsx
- Supplementalfigure1final.pdf
- Supplementalfigure2.pdf
- Supplementalfigure3.pdf
- Supplementalfigure4final.pdf
- Supplementalfigure5final.pdf
- Supplementalfigure6final.pdf
- Supplementalfigure7final.pdf
- SupplementalFigure8final.pdf

## Data Availability

The ST data from two AC, two PART, and two AD samples, along with the snRNA-seq data generated in this study, will be deposited in the GEO database upon manuscript acceptance. The raw FASTQ data have been deposited in the SRA database and are accessible for download through the accession number (PRJNA1300973). The public snRNA-seq data on human hippocampus were from ROSMAP project (syn52293442).

## CODE AVAILIABILITY

The codes are available and can be downloaded from Github (https://github.com/Yungong1/Spatial-Transcriptome-Hippocampus).
